# Preparing for COVID-19: early experience from an intensive care unit in Singapore

**DOI:** 10.1186/s13054-020-2814-x

**Published:** 2020-03-09

**Authors:** Mei Fong Liew, Wen Ting Siow, Graeme MacLaren, Kay Choong See

**Affiliations:** 1 Division of Respiratory and Critical Care Medicine, University Medicine Cluster, National University Health System, 1E Kent Ridge Rd, Singapore, 119228 Singapore; 2 Fast and Chronic Programmes, Alexandra Hospital, National University Health System, Singapore; 3 Cardiothoracic Intensive Care Unit, National University Health System, Singapore

Dear Editor,

About a third of patients infected with severe acute respiratory syndrome coronavirus 2 (SARS-CoV-2) become critically ill and require intensive care unit (ICU) admission [1]. As the COVID-19 (coronavirus disease-19) outbreak spreads [2], ICUs outside of China need to prepare for a potential surge of critically ill patients and counter the high transmissibility of SARS-CoV-2 [3]. Liu et al. have described their important preparations [4], and we would like to expand on their good advice by sharing lessons learnt from our early experience.

By 17 February 2020, Singapore recorded the highest number of confirmed cases outside of mainland China with several clusters of local transmission. All healthcare institutions adopted a common strategy of containment, with isolation of all suspected or confirmed cases of COVID-19 in negative-pressure rooms. We were fortunate that most ICU beds were single rooms—this infrastructure was put in place following the outbreak of SARS in 2003.

We realized preparing ICUs for patients with COVID-19 had numerous other requirements. First, infection control not only involved strict adherence to personal protective equipment for the individual, but also involved changes in group dynamics. We organized our ICU to mitigate the effects of any infected staff by avoiding potential spread between teams (see Table 1). Related to infection control, the medical ICU was given the task to cohort suspect or confirmed cases, including with peri- and post-partum care of pregnant women. Second, evolving information necessitated rapid and regular communications with large, disparate groups of clinicians.

**Table 1:** Critical care issues and solutions for COVID-19

Issues	Principles	Solutions
**Infection control**	1. Avoidance of cross-contamination among HCW 2. Education and re-education on personal protective equipment and use of powered air-purifying respirators 3. Provision for workflows to cater to special groups, such as pregnant women with acute respiratory illness who are in labour 4. Enhanced surveillance for infection in HCW 5. Strong emphasis on good hand hygiene for all 6. Robust visitor screening and management	• A dedicated roster to segregate “clean” and isolation teams, and to provide for stand-bys
		• Provision of clean scrubs for HCW to change into before duty; showering facilities at the end of shift
		• Education and re-education on personal protective equipment and use of powered air-purifying respirators, especially for isolation teams
		• Allow isolation teams to have a 2-week off-duty observation period (“wash-out” period), after every period of ward cover if manpower allows
		• Mandatory reporting of twice daily temperature monitoring by all HCW
		• Advance declaration of leave and overseas trips by HCW
		• Screening questions are regularly updated as case definitions evolve over time, especially for known clusters of infection in the community
		• Provision of thermal scanners at the doorstep to screen for fever
		• Maintaining a hospital visitor log to allow for contact tracing and activity mapping of confirmed cases
**Dissemination of information to HCW**	1. Robust system of dissemination of information (changing policies, workflows, etc.) 2. Email and meetings alone are insufficient to operationalize urgent changes on the ground 3. Clinical discussions of confirmed cases within the ICU community	• Utilization of secure and approved platforms such as institutional email and messaging applications to inform various job groups and teams of rapidly evolving workflows and policies
		• Utilization of secure videoconferencing applications to hold inter-institution and inter-department meetings and educational sessions
		• Utilization of secure and approved applications such as messaging and videoconferencing applications to conduct clinical discussions of cases and the sharing of experience
**Resuscitation and code blue response**	1. Provide clear guidelines on personal protective equipment and use of powered air-purifying respirators in ISO wards and normal wards during resuscitation 2. Provide inter-professional simulation of resuscitation scenarios for suspected or confirmed cases	• Simulation practice with personal protective equipment and use of powered air-purifying respirators will help identify gaps in the wards and prepare ISO teams for such scenarios
		• Simulation with limited team members per scenario, for example, 4 members per team, to allow acclimatization of HCW to perform resuscitation in smaller teams
		• Checklists for preparation of drugs and pre-prepared trolleys for equipment, for intubation, line setting and other procedures, to minimize staff movement and enhance efficiency
		• Creative ways to improve communications during resuscitation, such as utilization of a printed “Call Airway Team” card for difficult intubations, using a communication whiteboard in the patient room and using walkie-talkies to relay messages to staff outside the room for equipment and help
**Advanced ICU services**	1. To provide clear thresholds for transfers of deteriorating cases for ECMO 2. To provide efficient and safe delivery of ICU bronchoscopy	• Early transfer of deteriorating cases is recommended. Provision of thresholds for transfer and workflows for non-ECMO centres
		• Use of disposable bronchoscopes for bronchoscopy and percutaneous tracheostomy
**Psychological stress and burnout of HCW**	1. To provide emotional support, encouragement and appreciation to HCW 2. Reduce stigmatization of HCW by ill-informed members of the public	• Special provision of meals and drinks to boost morale; laundry service for used scrubs
		• Provision of regular updates of the local situation and status by the government and institution leadership
		• Frequent encouragement of HCW by divisional heads and senior leaders via emails, messaging apps and social media platforms, allowing staff to remain engaged
		• Timely articles and courageous stories of frontline staff
		• Appropriate media coverage of HCW at the frontline to increase empathy and reduce stigmatization

*ECMO* extracorporeal membrane oxygenation, *HCW* healthcare workers, *ICU* intensive care unit

Third, we had to train non-ICU acute medical staff dealing with critically ill patients prior to ICU admission, especially for resuscitation. Fourth, we had to re-examine specific ICU services. Given that extracorporeal membrane oxygenation (ECMO) for severe viral pneumonia is well-established [5], we prepared to cohort all COVID-19 patients in the medical ICU and have a satellite team from the cardiothoracic ICU manage the ECMO circuit. Lastly, we realized staff morale took an early hit due to multiple factors, including increased workload due to implementation of strict infection control measures, uncertainty over the effectiveness of personal protective equipment, anxiety over the lethality of any infection, concern for the well-being of their family members and stigmatization by members of the public.

To address the various issues of infection control, information flow, resuscitation training, advanced ICU services and psychological well-being of staff, we formulated several principles and solutions, which we hope can help other ICUs prepare for COVID-19 (see Table 1).

## Data Availability

Not applicable
